# Brief Acceptance and Commitment Therapy for Fibromyalgia: Feasibility and Effectiveness of a Replicated Single-Case Design

**DOI:** 10.1155/2020/7897268

**Published:** 2020-10-17

**Authors:** María Camino Gómez-Pérez, Azucena García-Palacios, Diana Castilla, Irene Zaragozá, Carlos Suso-Ribera

**Affiliations:** ^1^AMIMET, Tudela, Navarre, Spain; ^2^Department of Basic and Clinical Psychology and Psychobiology, Jaume I University, Castellón de la Plana, Spain; ^3^CIBER of Physiopathology of Obesity and Nutrition (CIBERobn),CB06/03, Instituto de Salud Carlos III, Madrid, Spain; ^4^Department of Personality, Evaluation and Psychological Treatment, University of Valencia, Valencia, Spain

## Abstract

**Objective:**

Overall, the literature on the effectiveness of psychological treatments in general and those for fibromyalgia in particular has been dominated by research designs that focus on large groups and explore changes on average, so the treatment impact at the individual level remains unclear. In this quasi-experimental, replicated single-case design, we will test the feasibility and effectiveness of a brief acceptance and committed therapy intervention using ecological momentary assessment supported by technology.

**Methods:**

The sample comprised 7 patients (3 in the individual condition and 4 in the group condition) who received a brief, 5-week psychological treatment. Patient evolution was assessed one week prior to treatment onset and during the whole study with a smartphone app. Because ecological momentary assessment and the use of an app are not frequent practices in routine care, we also evaluated the feasibility of this assessment methodology (i.e., compliance with the app). Change was investigated with a nonoverlap of all pairs index. Outcomes were pain interference with sleep and social activities, fatigue, sadness, and pain intensity.

**Results:**

Patient change was not uniform across outcomes. Four patients (two in each condition) showed relatively moderate levels of change (approximately 60% nonoverlap in several outcomes). The remaining patients showed more modest improvements which affected a reduced number of outcomes. Based on nonoverlapping indices, there was no clear evidence in favor of any treatment format.

**Conclusions:**

An alternative design to large-scale trials, one that focuses on the individual change, exists and it can be implemented in pain research. The use of technology (e.g., smartphones) simplifies such designs by facilitating ecological momentary assessment. Based on our findings showing that changes were not homogeneous across patients or outcomes, more single-case designs and patient-centered analyses (e.g., responder and moderation analyses) are required.

## 1. Introduction

Fibromyalgia (FM) is a rheumatic disease characterized by a history of widespread musculoskeletal chronic pain, sleep disturbance, debilitating fatigue, joint stiffness, and cognitive (memory, attention, perception, coordination) dysfunction and is frequently associated with depression and anxiety-related disorders [1–3]. FM is a very prevalent disease worldwide, with estimates suggesting that between 2% and 5% of individuals, mostly females, experience this syndrome globally [4, 5].

There is now quality evidence supporting the utility of several pharmacological (i.e., antidepressants and antiseizure drugs) and nonpharmacological treatment (i.e., Cognitive Behavioral Therapy, CBT) options for FM [6, 7]. Indeed, CBT has become a frequent treatment for FM patients, and systematic reviews and meta-analyses so far indicate that moderate effects on physical disability and mental distress are obtained with CBT, while pain intensity changes tend to be smaller [8, 9].

While these results with CBT are encouraging, the past years have evidenced an increasing interest in the so-called “third wave” psychological interventions for pain management, such as Acceptance and Commitment Therapy (ACT). Different from CBT, ACT does not aim at reducing or modifying distressing pain-related thoughts, emotions, sensations, or memories but promotes the psychological acceptance of suffering as a way of reaching personal values [10, 11]. Some authors have proposed that, due to the difficulties in controlling pain in chronic pain populations, including FM patients (e.g., note that the poorer effects of CBT on outcomes appear when attempting to reduce pain levels), ACT might better adjust to the needs of these patients because the goal lies in accepting suffering (i.e., pain), as opposed to trying to control it, in the process of leading a value-driven life [12, 13].

So far, the results on the effectiveness of ACT for FM are encouraging [14]. However, the results about the effectiveness of ACT in FM are mostly based on group studies with a limited number of assessments (i.e., pretreatment, posttreatment, and follow-up measures) [15, 16]. This means that there are important questions that remain unexplored. For instance, it is unclear whether a group format is more effective than an individual one for this population. Moreover, while the use of large samples and average change-scores in randomized controlled trials is a frequent practice in clinical research, these designs only show changes on average, so the effectiveness of ACT for FM at the individual level is yet unclear (i.e., for how many patients treatment works and to what extent). Additionally, these designs require the existence of a control group, which is ethically problematic. There is indeed a growing call for studies using research designs that overcome these limitations [17]. It has been argued that single-case designs (SCDs) are the preferred alternatives to large-scale randomized controlled trials, both in clinical research in general [18] and in chronic pain in particular [19, 20], due to their focus on the individual and the fact that no control group is required (patient baseline is used as the control condition).

In light of the previous limitations, in the present study we will compare the effectiveness of a brief ACT intervention for patients participating in a group or an individual treatment using a SCD. Contrary to the traditional episodic assessment of randomized clinical trials and in accordance with requirement for SCDs, assessment will be ecological and momentary using a smartphone app developed and validated by our team [21]. Treatment effectiveness will be investigated at the individual level. Based on previous research comparing group and individual CBT treatment in other populations [22], we expect to obtain similar effects in both formats as revealed by a reduction of pain interference in sleep and social activities, fatigue, and sadness. A reduction in pain intensity as a secondary gain of the intervention is also anticipated, but not primarily targeted. As recommended, the analyses of treatment effectiveness will not be explored visually, but using nonoverlap indices (i.e., nonoverlap of all pairs) [23, 24]. We expect to find similar nonoverlap indices (medium for pain interference with sleep and social activities, fatigue, and sadness and small for pain intensity) across patients, based on the assumption that group-level changes in past research are indicative of a similar improvement across individuals. These nonoverlap indices have become a recommended practice in SCDs as an alternative to visual analysis only as they allow obtaining more objective evidence of change by comparing the patient responses in the baseline against the scoring in the treatment phase [25].

## 2. Method

### 2.1. Design

A single-case AB (A: baseline; B: treatment) design was implemented. In psychological treatments designs, ABAB is not usually recommended, as it is not very usual that individuals return to baseline levels (A) after treatment (B) [26, 27]. Additionally, more robust designs, such as a multiple-baseline design, were not implemented here because they are difficult to use in group therapy (i.e., they require a stepped inclusion of the individuals in different sessions of the treatment) [28]. Therefore, while AB is a frequent SCD [29], it should be considered a quasi-experimental design. Therefore, causal inferences cannot be unequivocally made from the findings.

### 2.2. Procedures

Participants were recruited at the Fibromyalgia Association of Burriana and were treated at our University Psychological Assistance Service. Patients had been previously diagnosed with FM by a rheumatologist. The participants were randomly allocated to one of two conditions, (1) group therapy and (2) individual therapy, by an independent researcher using a randomization tool (http://www.randomizer.org). All participants gave their written consent to participate prior to randomization. An appointment was set one week before treatment onset to help participants download and make a first use of the app. This daily assessment during the week prior to treatment was used as baseline measurement. Participants continued using the app during the whole treatment (B). The study and assessment ended a week after the last treatment session (5 weeks after treatment onset).

Ecological momentary assessment was performed on a daily basis (twice a day, in the morning and in the evening, at preset times) with Pain Monitor, an app developed and validated by our team for chronic pain patients [21]. The app was used for 6 weeks (one week prior to treatment and five weeks during the treatment). Therefore, as recommended in the guidelines, at least five observations were obtained for each phase [17].

The study was approved by the Ethics Committee of the Jaume I University on June 24, 2018.

### 2.3. Sample

In SCDs, only 1 participant is required for the analyses. However, more participants are recommended for replication of effects and to facilitate the generalization of findings [17]. Therefore, we approached 13 patients at a FM association in Burriana (Spain), of which 10 agreed to participate, met the eligibility criteria, and provided written consent to participate. The resulting number of participants was 10 (5 for each condition). However, one participant from the group therapy did not receive treatment due to an unexpected surgical intervention happening just before treatment onset. Additionally, two patients in the individual therapy condition could not participate due to family problems that required their presence at home. Therefore, the final number of participants was 7 (4 in the group condition and 3 in the individual one).

The eligibility criteria included the following:

- The patient is over 18 years of age.
- The patient does not present with psychological and/or cognitive alterations or problems with language that make their participation difficult.
- The patient has the physical ability to use the application.
- The patient voluntarily wants to participate and signs the informed consent form.
- The patient has a mobile phone with an Android operating system.

In terms of eligibility, it is important to note that past research has indicated that the use of technology is often more problematic in the elderly [30]. This could lead us to think of old age as a potential exclusion criterion. However, our team has a long experience designing technologies for the elderly, and the application used in the present investigation has been successfully implemented in previous research with older patients with chronic pain [21]. Therefore, participation in the present study was not restricted to younger individuals despite the inclusion of a smartphone application for assessment, and participants were only deemed ineligible if they had physical problems that would prevent them from using the application.

### 2.4. Treatment Plan

The treatment program is a brief ACT protocol based on an ACT treatment created by our group, LabPsiTec, which has been used for FM in a group format in primary care settings [31]. Both treatments followed the same protocol and were delivered by the same psychologist, MC, who is experienced in the use of ACT in patients with FM. The use of such brief ACT interventions is becoming a frequent and recommended practice in clinical settings [32] and might be especially suitable for FM patients due to a frequent dropout rate and difficulties in attending treatment in this population, frequently as a consequence of their health status [15, 33].

The treatment included 5 weekly sessions. Each session was 1 hour long for the individual therapy and had a duration of 1 and a half hours for the group condition. Group therapies require a longer extension as the participants need more time to interact with each other [34].

As noted before, the number of sessions was reduced to obtain a brief version that includes the key aspects of ACT (i.e., psychological flexibility, acceptance of pain, cognitive defusion, values, mindfulness, and committed action) because long treatments have shown high dropout rates due to time constraints in this type of patients, which would be problematic in a single-case design.

Treatment started on 26/04/2018 and had a duration of 5 weeks. The treatment components are described in Table 1.

### 2.5. Measures

#### 2.5.1. Pain Monitor App

The application used in this study, Pain Monitor, can be downloaded for free at the Google Play store:

https://play.google.com/store/apps/details?id=painmonitor.srccode&hl.

The content in the app was developed by a multidisciplinary team of psychologists, physicians, and nurses from the Pain Clinic of the Vall d'Hebron Hospital and the LabPsiTec group of the Jaume I University. All items in the app have been adapted from well-established measures, and the complete protocol to be used in an app and EMA format has been recently validated in a chronic pain population [21]. The app uses a push system to inform the patient when to respond. Assessments occur twice a day, in the morning and the evening (10 am and 7 pm, with a two-hour flexibility). Study outcomes included pain interference with sleep and social activities, fatigue, and sadness (primary outcomes), as well as pain intensity (secondary outcome). The items used to measure the study outcomes are described in Appendix A. The focus on outcomes beyond pain intensity is consistent with modern perspectives on chronic pain management [35]. Most constructs in the app are assessed twice a day (e.g., because sadness can vary from morning to evening, this was evaluated at both times). However, pain interference was evaluated once a day only due to its content (e.g., because all participants slept at night, interference with sleep was evaluated in the morning only, while interference with social activities was assessed in the evening only). All items have been adapted from and validated against traditional, well-established scales in a previous study [21]. A reduced number of items is fundamental in ecological momentary assessment [36].

### 2.6. Calculations and Analyses

Traditionally, visual analysis of the data has been used as an indicator of performance in single-case research, by observing differences between the baseline (A) and the intervention (B) phases. However, visual analysis shows many limitations, including type 1 and 2 errors and poor interrater reliability [17]. Nonoverlap indices, specially the nonoverlap of all pairs (NAP) which is the more robust to bias, can solve some of the problems of visual analysis by offering a mathematical calculation of the overlap between A and B phases [24].

Different from other overlap indices, NAP accounts for all data overlap between each phase A data point and each phase B measurement [37]. NAP can be relatively easily calculated by dividing the number of nonoverlapping comparisons (between each A and B point) by the total number of possible comparisons. Therefore, NAP calculates the percentage of data that shows an improvement (functional outcomes) or a deterioration (dysfunctional outcomes) with respect to the baseline. In order to interpret the NAP values, the median nonoverlap of treatment studies has been proposed [38]. A nonoverlap below 38% corresponds to the 25^th^ percentile of lowest reported NAP scores across studies, so this should be interpreted as a poor treatment effect. A NAP between 38% and 68% would include studies between the 25^th^ and 50^th^ percentile of published effects, which might be interpreted as representing a mild-to-moderate intervention effect. A moderate-to-large effect would be represented by nonoverlap indices between 69% and 96% (median nonoverlap effects of studies between the 50^th^ and the 75^th^ percentile). Finally, NAP scores over 96% correspond to very large treatment effects (median effects of studies above the 75^th^ percentile of published effects). Overlap indices are calculated using within-person data and compare the baseline to all data points obtained during the entire treatment period.

Compliance with the app was explored both for morning and evening assessments and was calculated by dividing the number of reported responses by the number of possible assessments.

## 3. Results

Patient demographic characteristics, as well as their nonoverlap calculations, and app compliance rates are reported in Table 2.

### 3.1. Participants

All participants were women. Their age ranged between 53 and 67 years, and all of them had been suffering FM symptoms for more than 5 years. Educational level was low in most participants (primary or secondary education only, with the exception of a patient who had completed technical studies), and they were not very familiar with the use of smartphones applications. One woman was a widow, while the remaining participants were married.

### 3.2. Nonoverlap Analyses

Regarding interference with sleep, three patients (patients 1 and 2 from the group condition and patient 7 who followed an individual intervention) showed very poor improvement (less than 10% of nonoverlap). Their average baseline interference of pain with sleep was 8.8, 4.2, and 3.2, respectively. The remaining participants showed an improvement that corresponds to the 25^th^-50^th^ percentile range of existing effects in AB SCDs (i.e., between 48% and 60% of nonoverlap in our sample), which should be interpreted as mild-to-moderate effects [38]. Their average baseline interference of pain with sleep ranged from 3.2 to 5.6.

Different from interference with sleep, interference of pain with social functioning was more satisfactorily improved in the sample. Specifically, three patients (patient 2 in the group condition and patients 5 and 6 in the individual treatment condition) showed an improvement which is close to the 50^th^ percentile of existing effects in AB SCDs (i.e., between 60% and 62% of nonoverlap), which should be interpreted as a moderate effect. Their average baseline interference of pain with social functioning was 4.8, 1.4, and 2.0, respectively. Additionally, the remaining patients showed larger improvements compared to interference with sleep (i.e., between 22% and 35% nonoverlap). Their average baseline interference of pain with social functioning ranged from 3.6 to 5.0.

Regarding fatigue, only two patients in the group condition obtained nonoverlap scores that would correspond to less than the 25^th^ percentile of existing effects in AB SCDs. Baseline average fatigue levels of the two individuals were 6.7 and 6.2. The remaining patients showed improvements that would be between the 25^th^ and the 50^th^ percentile of the aforementioned effects (i.e., NAP between 41% and 53%), that is, changes that correspond to mild-to-moderate effects. Their average baseline fatigue levels ranged from 4.3 to 6.7.

In a similar way to fatigue, three patients (two in the group condition and one in the individual treatment condition) showed poor improvement in sadness (i.e., scores below the 25^th^ percentile). These patients had baseline sadness scores ranging from 4.33 to 5.57. The remaining patients showed larger improvements than those for fatigue (i.e., NAP between 53% and 64%), which would again correspond to NAP values between the 25^th^ and the 50^th^ percentile of existing effects in AB SCDs. Their average baseline sadness scores had a 2.78–6.89 range.

Finally, improvement in pain levels was poor (i.e., below the 25^th^ percentile of existing effects in AB SCDs) for most patients (i.e., four). Their average baseline pain levels ranged from 5.9 to 6.9. Three patients showed a NAP between 47% and 55%, which would correspond to NAP values between the 25^th^ and the 50^th^ percentile of existing effects in AB SCDs. Their average baseline pain levels were 3.7 (patient 6), 5.6 (patient 5), and 6.3 (patient 7).

**Figure 1:**
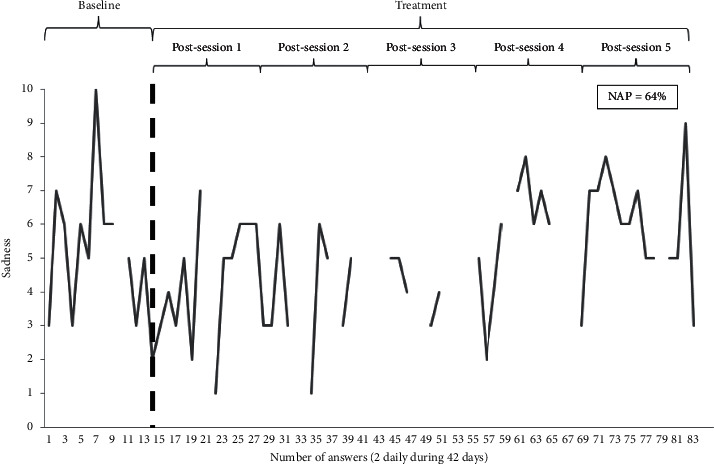
Patient 3 evolution in sadness (moderate improvement). NAP: nonoverlap of all pairs.

**Figure 2:**
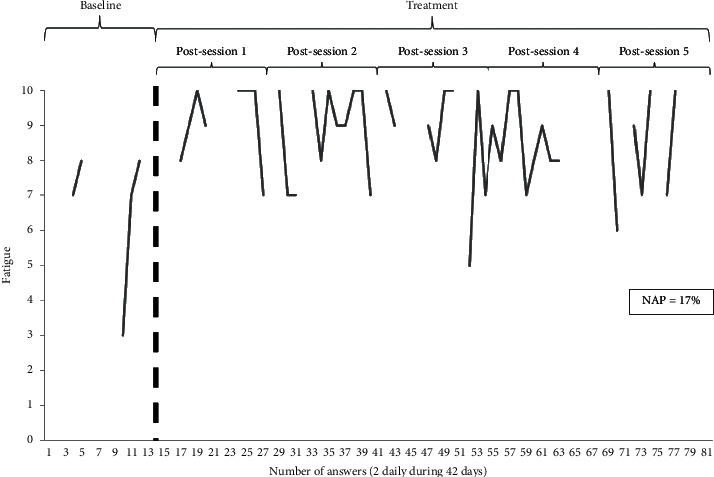
Patient 2 evolution in fatigue (no improvement). NAP: nonoverlap of all pairs.

Figures 1 and 2 show a graphical representation of a very poor and a modest response to treatment, respectively.

### 3.3. Feasibility of EMA Using an App

All patients reported at least five responses in each phase (baseline and treatment). At the group level, evening compliance with the app was 68.37% (participants provided 195 responses out of 294 requested measurements). Morning compliance with the app was 61.22% (participants provided 180 responses out of 294 requested measurements). As reported in Table 2, at the patient level, response rates ranged from 45.2% to 88.10%. Only one patient reported response rates below 50%, and this was due to technical problems with the phone (low memory capacity) that blocked the push system that reminds the patient to respond to the app. When this was identified (an alarm that reached the researchers was set to identify when a patient failed to respond to three consecutive assessments), we addressed this by encouraging the patient to respond to the app even in the absence of notifications, but this made compliance more difficult.

## 4. Discussion

The literature on clinical research in general and chronic pain in particular has been largely dominated by large-scale randomized clinical trials in which the analysis of changes, mostly with group interventions, is explored considering average group scores and a reduced number of assessments [39, 40]. While large-scale randomized clinical trials are clearly informative and can address very important questions including responder and moderation analyses, the overrepresentation of this type of studies in the literature can also be problematic for a number of reasons. First, because such designs mostly focus on average population treatments effects [41, 42]. Additionally, an alternative to large-scale trials is required for studies addressing the development of new treatments and the intervention with infrequent disorders, to test the efficacy of an intervention in routine practice with a reduced number of individuals. This study contributes to the existent literature in this direction by testing the effectiveness of a brief psychological treatment for chronic pain in routine care. First, it focused on changes at the individual level by implementing a single-case design. Additionally, it implemented a brief ACT treatment, which might be more feasible for FM patients in terms of adherence due to its short duration, and explored changes using a modern analytic strategy (i.e., nonoverlap of all pairs) which minimizes the reliability problems of visual analysis. Finally, the present investigation evaluated all outcomes ecologically and repeatedly (i.e., daily) using a modern approach, that is, a validated smartphone app. Thus, the present investigation might help inspire and serve as a guide for future investigations in pain research.

One of the findings in the present investigation was that not all patients improved to the same extent after treatment onset. Also interestingly, changes were not homogeneous across outcomes. For example, patients 5 and 6 showed relatively similar levels of change in all study outcomes. On the contrary, the majority of patients showed some change in a number of outcomes (e.g., patient 2 in social interference, patient 3 in sadness, and patient 7 in pain intensity) and barely no change in others (e.g., patient 2 in interference with sleep, fatigue, sadness, and pain intensity and patient 7 in sleep interference and sadness). As recommended in recent clinical research including the pain literature [27, 43], these findings suggest that aggregating data is indeed likely to mask a lack of response of an important number of individuals in some outcomes by including larger changes by other individuals in such variables. As noted many years ago, while managers and trialists might be happy when treatments work on average, patients surely expect clinicians to do better than that [44]. The present investigation is a step forward in this direction.

Another interesting result was that, based on the NAP indices, there was no clear evidence in favor of any of the two treatment conditions (group or individual intervention). This is consistent with our hypothesis based on a previous meta-analysis [22]. Some positive benefits have been traditionally attributed to group interventions, such as vicarious learning and social skills practice [34, 45], although individual treatments are still preferred by patients [46]. Interestingly, what the present and the aforementioned meta-analysis indicate is that individual and group interventions might lead to comparable benefits in FM populations. In fact, it is possible that certain formats are more adequate depending on patient characteristics than others (e.g., patient personality). In our study, for example, some patients showed acceptable changes after individual treatment (e.g., patients 5 and 6), while patient 7 showed very little change after treatment. A similar finding occurred with group treatment (i.e., overall, the changes were larger for patients 3 and 4 and smaller for patients 1 and 2). The reduced number of individuals in the present investigation prevents us from proposing any further conclusions in this regard, but this raises interesting questions for future research and for treatment personalization. While acknowledging this, it is also important to note that group interventions, if similarly effective, are often preferred by institutions because they can be more cost-effective [47], so efforts should be made to encourage patients to participate in group interventions due to the limited resources in public health settings [34].

As noted in the previous lines, not all patients responded to the intervention to the same extent. The analysis of predictors of response to treatment has been a topic of interest to researchers in different health fields for decades [48]. In chronic pain, however, research to date supports the idea that psychological treatment has a similar effect on patient outcomes regardless of patient baseline characteristics [49], even when the intervention is delivered online [50]. A formal moderation analysis was not performed in the present study due to the small number of participants and the complexity of the data (a combination of all outcomes, NAP indices, and baseline characteristics would be needed in a formal analysis of moderators). Nevertheless, a visual inspection of the data seems to support the aforementioned idea that baseline clinical and demographic characteristics do not moderate the response to psychological treatment. For example, patients 5 and 6, who generally responded well to the intervention, had somewhat different levels of pain (5.6 and 3.7, respectively). Furthermore, pain levels were similar in patients who had an acceptable response to the treatment (3 to 6) and in patients who responded poorly to the intervention (patients 1, 2, and 7). More research on moderators of response to treatment is needed [51], for example, by including psychological characteristics such as fear-avoidance beliefs and pain catastrophizing [52, 53].

Still, in relation to the effectiveness of treatment, the present investigation showed that ACT was less effective in reducing pain severity compared to the remaining outcomes. Research on FM has already indicated that changing pain levels with psychological therapy is more challenging than addressing other outcomes, such as depressive symptoms and functional status [54–56]. According to ACT, a reduction in pain is not necessary to improve functioning [57]. Therefore, the present study findings indicating that outcomes other than pain severity were improved to a greater extent are consistent with ACT principles.

In addition to the exploration of treatment effectiveness, an important study goal was to explore the feasibility of implementing a new way of tracking patient response to treatment, that is, EMA using a smartphone app. Overall, the response rates suggest that the use of this new assessment method is feasible, although app compliance was slightly lower than that in similar studies [21]. However, it is important to note that the population in the present investigation was older and less educated than that of the previously mentioned study, which might have negatively influenced the participants' ability to comply with EMA using technology. In fact, the participants were generally not very familiar with the use of technology. Additional factors that might also have contributed to lower response rates in the present investigation are the duration of EMA, which was two weeks longer in the present study, and the type of pain in the populations, which was mostly back and neck pain in the aforementioned investigation as opposed to fibromyalgia in the present study. Indeed, the use of technology appears to be more challenging in the elderly [30]. However, as noted earlier in the present study, the use of smartphone applications for patient monitoring has important advantages over traditional paper-and-pencil diaries and episodic assessment, which helps understand why this method has become the gold standard in health settings [58]. It is expected that, with the growing availability of smartphones and their widespread utilization globally and across ages, we will see a reduction in technological barriers in the elderly in the coming years [59].

Regarding app compliance, it is also important to note that evening compliance was higher compared to morning response rates. This is consistent with past findings [21] and might be explained by the fact that sleep quality in this population is frequently poor and often impacts on morning functioning status [60]. While job status has been argued to explain higher response rates in the evening (e.g., some individuals working in the morning might have difficulties in completing morning reports at work) [21], only one participant in the present study was working during the study. Thus, morning-to-evening differences in assessment completion rates are not likely to be explained by job status. Future research should take this into account maximizing compliance with EMA in this population (i.e., by delaying morning responses, which were planned to be at 10 am in the present study, according to the patients' preferred hours).

Strengths of the present investigation include the implementation of a single-case design with several replications and the implementation of technology (i.e., a smartphone app) for daily monitoring. Previous single-case investigations in pain research have either used paper diaries for daily assessment [43, 61], which are known to be frequently completed retrospectively [62], or investigated the impact of ACT on a single patient using two measurement points only, that is, pretreatment and posttreatment [63], which does not allow establishing a reliable picture of patient baseline scores and change after treatment. In fact, the study figures clearly proves the need for ecological momentary assessment in an attempt to obtain a more reliable measurement of outcomes in FM patients, thus supporting the idea that ecological momentary assessment using technology is the gold standard monitoring method in pain [64]. Therefore, the present investigation might provide researchers with a novel approach to exploring patient evolution and treatment effectiveness in pain settings as an alternative to episodic, on-site assessment in large-scale randomized controlled trials.

Despite the previous strengths, some study limitations should also be considered. First, the study design was quasi-experimental. While the reasons for not selecting more complex, experimental SCDs have been already explained (i.e., the comparison of group and individual treatment would make multiple baselines complicated, and the implementation of a psychological intervention would make ABAB designs inadequate), this affects the internal validity of findings (i.e., causality cannot be claimed). Additionally, while replication was made for external validity, the number of replications should be increased to allow for more complex analyses (i.e., to explore whether a given format is more effective for certain individuals or whether the extent to which change occurs in a given patients can be related to personal characteristics). These are interesting lines of research which might provide the reader with novel ideas for future pain studies. Additionally, note that only 3 patients were retained in the individual treatment condition, while the best practice guidelines recommend 4 participants for replication. Finally, the patient response rate was lower than intended. While this provided sufficient information for our analyses, the results are not ideal. It is possible that the reasons are their low level of education, their poor experience with new technologies, and the technical problems experienced with some old smartphones (i.e., low internal memory in some moments which blocked the push system), so our experience should serve similar studies to anticipate these problems.

While acknowledging these limitations, the present study might also have some important clinical and research implications. First, it proved that a novel approach to pain research, one that puts the focus on the patient as opposed to average group scores, is possible. The pain literature on ACT and other psychological interventions is clearly dominated by randomized controlled trials which provide information of average group response to treatment and evaluate outcomes episodically [65, 66]. It is time to explore alternatives to such designs, and the present study is a first step in this direction.

## Figures and Tables

**Table 1:** Psychological components of the program.

Session	Component	Goal
1	Psychoeducation	Brief description of the most prominent symptoms of FM and their interference in the daily activities and emotional functioning. In the group condition, it also included rules and structure
	Motivation for change	Motivation for changing our behavior patterns
2	Acceptance	Learning to be in contact with internal bodily sensations (i.e., pain), even if they are unpleasant. Willingness to accept them as they are in the way of achieving a more meaningful life
	Mindfulness	
	Cognitive defusion	Experiencing sensations as they are and not the way the mind says they are
	Self as observer	Identification of the observer self
3	Values	Clarification of personal, professional, and health-related values
	Committed action	Taking value-driven actions
4	Compassion	Learning self-care techniques (compassion)
5	Relapse prevention	Revision of acquired abilities
		Anticipation of future difficulties and barriers and detection of skills to be used in such scenarios

**Table 2:** Demographic characteristics, nonoverlap calculations, average baseline scores, and compliance with the app.

Patient	Demographic characteristics			Treatment condition	Interference with sleep		Interference with social activities		Fatigue		Sadness		Pain intensity		Compliance with the app	
	Age	Education	Job status		NAP (%)	Baseline	NAP (%)	Baseline	NAP (%)	Baseline	NAP (%)	Baseline	NAP (%)	Baseline	Morning (%)	Evening (%)
1	67	Primary	Homemaker	Group	9	8.8	33	4.2	49	4.4	20	4.3	25	5.9	59.5	57.1
2	65	Primary	Homemaker	Group	2	4.2	62	4.8	17	6.7	13	5.6	13	6.1	69.0	69.0
3	53	Primary	Working	Group	48	5.6	31	5.0	35	6.2	64	5.2	38	5.9	57.1	61.9
4	54	Secondary	Unemployed	Group	50	5.0	22	4.8	47	7.8	58	6.9	38	6.9	57.1	64.3
5	67	Primary	TPD	Individual	49	3.2	62	1.4	41	5.9	53	2.8	55	5.6	66.7	88.1
6	66	Primary	Homemaker	Individual	60	5.0	60	2.0	53	4.3	61	3.7	47	3.7	45.2	59.5
7	62	Secondary	TPD	Individual	0	3.2	35	3.6	42	6.3	27	5.4	51	6.3	73.8	78.6

NAP: percentage of nonoverlap of all pairs; TPD: total permanent disability. Compared to previously reported nonoverlap indices [38], results should be tentatively interpreted as follows: <38%: poor effect; 38% to 68%: mild-to-moderate effect; 69% to 96%: moderate-to-large effect; >96%: very large effect. Baseline scores refer to the average scores from the first week before treatment onset.

## Data Availability

The data used to support the findings of this study are available from the corresponding author upon request.

## Supplementary Materials

Supplementary Materials Appendix A: items used in the Pain Monitor app to measure the study outcomes.
