# Supplementary material for: Brief Acceptance and Commitment Therapy for Fibromyalgia: Feasibility and Effectiveness of a Replicated Single-Case Design
Source: Pain Res Manag. 2020 Oct 17;2020:7897268. doi: 10.1155/2020/7897268 (PMC7586182; doi:10.1155/2020/7897268)
Supplement: Supplementary Materials — Appendix A: items used in the Pain Monitor app to measure the study outcomes. [file 7897268.f1.docx]

**Appendix A: Items used in the Pain Monitor app to measure the study outcomes**

*Pain interference on sleep:*

Did your PAIN interfere with the quality of your SLEEP LAST NIGHT?

0 No interference ------- 10 Maximum interference

*Pain interference on social activities:*

Did your PAIN interfere with your SOCIAL INTERACTIONS TODAY?

0 No interference ------- 10 Maximum interference

*Fatigue:*

Please indicate the intensity of your CURRENT FATIGUE:

0 No fatigue ---------10 Extreme fatigue

*Sadness:*

Please indicate the intensity of your CURRENT SADNESS:

0 No sadness -------- 10 Extremely sad

*Pain intensity:*

Please indicate the intensity of your CURRENT PAIN:

0 No pain ---------10 Extreme pain
